# Regenerative Strategies in Treatment of Peripheral Nerve Injuries in Different Animal Models

**DOI:** 10.1007/s13770-023-00559-4

**Published:** 2023-08-12

**Authors:** Mona M Khaled, Asmaa M Ibrahium, Ahmed I Abdelgalil, Mohamed A. El-Saied, Samah H El-Bably

**Affiliations:** 1 Department of Anatomy and Embryology, Faculty of Veterinary Medicine, Cairo University, Giza Square, Giza, 12211 Egypt; 2 Department of Surgery, Anaesthesiology and Radiology, Faculty of Veterinary Medicine, Cairo University, Giza Square, Giza, 12211 Egypt; 3 Department of Pathology, Faculty of Veterinary of Veterinary Medicine, Cairo University, Giza Square, Giza, 12211 Egypt

**Keywords:** Peripheral nerve injuries, Sciatic nerve, Stem cells, Nerve regeneration, And Schwann cells

## Abstract

**BACKGROUND:**

Peripheral nerve damage mainly resulted from traumatic or infectious causes; the main signs of a damaged nerve are the loss of sensory and/or motor functions. The injured nerve has limited regenerative capacity and is recovered by the body itself, the recovery process depends on the severity of damage to the nerve, nowadays the use of stem cells is one of the new and advanced methods for treatment of these problems.

**METHOD:**

Following our review, data are collected from different databases "Google scholar, Springer, Elsevier, Egyptian Knowledge Bank, and PubMed" using different keywords such as Peripheral nerve damage, Radial Nerve, Sciatic Nerve, Animals, Nerve regeneration, and Stem cell to investigate the different methods taken in consideration for regeneration of PNI.

**RESULT:**

This review contains tables illustrating all forms and types of regenerative medicine used in treatment of peripheral nerve injuries (PNI) including different types of stem cells " adipose-derived stem cells, bone marrow stem cells, Human umbilical cord stem cells, embryonic stem cells" and their effect on re-constitution and functional recovery of the damaged nerve which evaluated by physical, histological, Immuno-histochemical, biochemical evaluation, and the review illuminated the best regenerative strategies help in rapid peripheral nerve regeneration in different animal models included horse, dog, cat, sheep, monkey, pig, mice and rat.

**CONCLUSION:**

Old surgical attempts such as neurorrhaphy, autogenic nerve transplantation, and Schwann cell implantation have a limited power of recovery in cases of large nerve defects. Stem cell therapy including mesenchymal stromal cells has a high potential differentiation capacity to renew and form a new nerve and also restore its function.

## Introduction

Traumatic affections of peripheral nerves were the most common serious problems leading to a long-lasting disability including sensory and motor dysfunction, neuropathic pain, muscle atrophy, and even limitation of life [1]. Sciatic, peroneal, tibia, brachial plexus, and radial nerves were the most commonly affected peripheral nerves [2].

The radial nerve is the largest nerve of the brachial plexus supplying all extensors of carpal and digit and even sensation of dorsal region of carpus and digit, its injury occurs as a result of drag, transaction, pressurization, vehicle accident that leads to a complete humeral fracture and also local injections of some drugs [3].

Sciatic nerve is the thickest nerve in the whole body and it is considered the main direct continuation of lumbosacral plexus, descending as distal as the heel of the foot, supplies several muscles in the leg and even sensation of most skin of the lower leg [4–6]. Sciatic injuries were commonly caused by compression, stretching or traction, laceration, crushing, and also pelvic fracture [1].

Central nervous system (CNS) has no tendency to heal, while peripheral nerve repair is limited, and complete recovery doesn't occur in most critical conditions. Repairing mechanism is a complex of several pathological interactions such as neuronal and axonal regeneration, Wallerian degeneration, production of inflammatory cytokines, and neurotrophic factors from Schwan cells [7].

Traditionally, Peripheral nerve injuries (PNI) have been managed surgically either by end-to-end or end-to-site anastomosis. Nerve auto-grafting in long nerve defects was considered the gold standard for management of PNI but has some restrictions for obtaining donor nerve, donor –nerve infection, and neuroma formation [4]. Schwan cell auto transplantation has two major disadvantages including a long time for *in vitro* growth and culture and also the additional damage to donors [8].

Besides the disadvantages of the previous traditional methods, they don't achieve full nerve recovery and a satisfactory result has not been obtained. Nowadays, medicine is directed to tissue engineering to solve most regardless cases of nerve dysfunction, organ failure, and end-stage disease [7]. Transplantation of allogenic Schwan cells together with a nerve scaffold helps in provoking therapeutic nerve repair and become an applicable method in human cases [4].

Mesenchymal stem cells (MSCS) are multi-potent, plastic, un-differentiated cells that can be obtained from several body sources such as bone marrow, adipose tissue, amniotic membrane, dental pulp, and umbilical cord [9]. MSCS transplantation has proved a successful progress in repairing most damaged tissues and this capacity came from their self-renewal, fast-proliferation, and multi-potent differentiation [8]. Bone marrow mesenchymal stem cells (BMSCS) promote nerve repair through synthesis of neurotrophic factors such as" nerve growth factor (NGF), brain-derived neurotrophic factor (BDNF), neurotrophin 3 (NTF3), and glial cell-derived neurotrophic factor (GDNF) ". The BMSCS isolation is considered an invasive technique with a low number of harvested cells to enhance proliferation and differentiation [8]. So, trials are directed toward adipose-derived stem cells which have the same phenotype and genotype regeneration power as BMSCS but rejoice by other features as it can be easily obtained from any fat-rich source, its low immunogenicity, and its faster proliferation than BMSCS [10].

Nerve guidance conduits are tubular structures that can be used for bridging and regeneration of nerve gap defects when injected with extracellular matrix proteins (ECM), growth factors, or supporting cells when autografting is limited due to donor availability [11–13]. There were different kinds of artificial nerve conduits such as chitosan, collagen, and poly (DL-lactide-ε-caprolactone) and they are considered the most commonly and frequently used in research studies [14, 15].

The properties of the ideal nerve conduits were good biodegradability and biocompatibility within the tissue, better porosity and permeability for drug release, and low immunogenicity and toxicity for the body. These characteristics facilitate neural regeneration and stimulate axonal remyelination at the site of injury [16, 17]. In our review, we demonstrate various nerve conduits used for the repair of nerve injury and the potential effect of each type in peripheral nerve regeneration.

ADSCs are pluripotent adult stem cells, obtained from any fat-rich sources that had the properties of rapid proliferation and multi-lineage differentiation into osteoblasts, chondrocytes, and adipocytes as BMSCs, but it differs in their higher intensity in fatty tissue after isolation, so it takes a shorter period for tissue regeneration than BMSCs. ADSCs have low immunogenicity power and are rarely rejected by the recipients [8, 18, 19].

BMSCs are another multipotent adult mesenchymal stem cells that can be easily obtained through aspiration of bone marrow. They can differentiate into bone, cartilage, and fat cells and present neural or glial markers typical of Schwann cells in several neurodegenerative diseases. However, they have low proliferation power than embryonic stem cells and a more invasive method than ADSCs as they require anesthesia in addition to their low cell count after isolation regarding other types of stem cells [20–22].

EPCs (Endothelial progenitor cells or umbilical cord mesenchymal stem cells) are proliferative cells produced from fetal tissue after birth with no invasive procedure to the donor or patient and are also available in cell banks. They can be easily and ethically obtained compared with embryonic or bone marrow stem cells, so they are usually preferred over other types. The proliferation capacity and differentiation power of umbilical cord mesenchymal stem cells are proved to be higher when compared with bone marrow mesenchymal stem cells [18, 23, 24].

## Material and method

Our review collected from different databases focusing on research investigating the different treatment strategies of peripheral nerve injuries including therapeutic medicines, nerve conduits, and different types of stem cells in different animal models (rat, mice, rabbit, cat, dog, monkey, pig and sheep hoping that these trials can be used as an "off-shelf" medicine in human and animal application.

## Result

### Peripheral nerve injury (PNI)

Stretch-related injuries are the most common and frequent type of PNI, and their etiology is usually inherited where the nerve nature is very elastic due to its collagenous endoneurium. The nerve injury occurs when it is exposed to a force exceeding its strength and when this force is high enough may result in a full disruption of nerve continuity as in brachial plexus avulsion. Stretch-related injuries occur in the case of nerves that are anatomically related to bones as radial nerve paralysis in complete humeral fracture [25].

The second common type is laceration injuries, which represent about 30% of serious cases, whereas a complete cutting of the nerve or parts from it occurs. This type is mostly involved in research because it is easier to be performed [26].

The third type of PNI is nerve compression which may be induced either by mechanical deformation or ischemia. The most severe form of this type is related to complete disruption of the action potential without any tearing or transection of nerve fibers. So, researchers confused about the pathophysiology of this type either is due to external pressure applied on the nerve or due to induced ischemia especially since there is no, or little histological evidence reported in this type. “Saturday Night palsy” due to radial nerve compression was documented and ultra-structural studies showed myelin and axoplasm dis-positioning and nerve fibers degeneration at the compression site more severe than in areas away from the compression [27].

### Nerve injury classification and grading

Seddon divided nerve injuries by severity into three broad categories: neurapraxia, axonotmesis, and neurotmesis. Neurapraxia is the simplest form of injury with a mild interruption in nerve impulse conduction, but the continuity of the nerve is not affected. So, it is a transient condition with varying recovery periods. Axonotmesis means discontinuity in axon and myelin sheath with damaged endoneurium and perineurium. Neurotmesis involves disconnection of a nerve with loss of nerve function and recovery without surgical intervention does not usually occur because of scar formation and the loss of the mesenchymal guide that properly directs axonal regrowth [28].

Sunderland’s classification system further re-classifies three injury types described by Seddon into five categories depending on the severity of the damage as shown in Table 1 A first-degree injury is equivalent to Seddon’s neurapraxia, and a second-degree injury is equivalent to axonotmesis. Third-degree nerve injuries occur when there is disruption of the axon (axonotmesis with partial loss in endoneurium). Seddon’s neurotmesis represents fourth- and fifth-degree injuries in Sunderland’s classification. In a fourth-degree injury, all parts of the nerve are damaged except the epineurium. Five-degree injury is the most severe form in which there is complete dis-connection of the nerve [29].

**Table 1:** Classification of nerve injury according to Sunderland and Seddon (1943)

Sunderland	Process	Seddon
Classification of nerve injury		
First degree “G1”	Slight contusion on the nerve results in an interruption in transmission of impulse Although the whole anatomy of neuron remains the same, the affected region suffered from a reduction in impulse transfer. Recovery from this type takes place within 3 months [5], or 3-6 weeks [6] “Segmental demyelination”	Neuropraxia
Second degree “G2”	Damage to axon with removal of Wallerian sheath but Schwan cell & endoneurium still intact	Axontmesis
Third degree “G3”	Endoneurium is partially compressed but perineurium and epineurium are still intact	Axontmesis
Fourth degree “G4”	Endoneurium and perineurium are damaged but epineurium is still intact	Neurotmesis
Fifth degree “G5”	Complete anatomical disruption in nerve continuity, with fibrous tissue proliferation, damaged endoneurium, perineurium, and epineurium & no nerve recover [5]	Neurotmesis

### Neuropathology and mechanism of injury

The peripheral nerve trunk is mainly composed of several nerve fascicles and is surrounded by its connective tissue sheath known as epineurium. Each fascicle consists of groupings of nerve axons entrapping within the endoneurium sheath while the nerve fascicles are surrounded by a different type of sheath called perineurium. The orientation of fibers in each sheath differs from each other, the perineurium and epineurium are circular but the endoneurium is longitudinally aligned. Many blood capillaries are distributed within the epineural sheath and give collateral minor vessels to supply the endoneurial sheath through the perineurium. This vascularization system can aid in introducing a secondary injury to the nerve when expose to severe compression leading to vascular edema causing additional pressure on the nerve structure and contribute to nerve trauma [30].

There are various mechanisms for applying pressure on the nerve and succeeding in nerve injury. Compression of vascular capillaries supplying peripheral nerve when the nerve runs through a narrow anatomical position causes ischemia to the nerve and is categorized as grade 1 injuries or neuropraxia. Traumatic compression by a blunt object as surgical forceps or clamps to an extended period without nerve cutting is usually categorized as a crush injury. Laceration by a knife, gunshot, or glass piece led to the discontinuation of the nerve known as neurotmesis [31].

Sciatic and radial nerve damage may be occurred after intra-muscular injection due to the harmful effect of the injected drug, or the physical trauma caused by in correct method of injection by a less experienced person resulting in sever trouble shock in sensation and may extend to a motor dysfunction [32].

### Neural response to injury

A series of degenerative cascades occur after nerve injury which is considered a direct precursor to regeneration. The extent of regeneration is primarily influenced by the degree of initial damage and subsequent degenerative changes. In first-degree, there is only a conduction block and no true degeneration or regeneration; pathological changes are mild or nonexistent. In the second degree, a calcium-mediated process known as Wallerian (or anterograde) degeneration occurs distal to the injury site with little histological change at the injury site or nearby [33].

In third-degree injuries (intra fascicular injuries), a significant trauma-induced local reaction displaces. The elastic endoneurium retracts the ends of severed nerve fiber. Hemorrhage and edema from local vascular trauma cause a strong inflammatory response. The injured segment develops a dense fibrous scar as fibroblasts multiply, which results in fusiform swelling (neuroma) and additional perineural scar tissue [25].

### Treatment strategies involved in PNI

In recent years, most candidates are directed to advanced alternative medicines in manipulation of PNI cases instead of previous old surgical interventions like end-to-end anastomosis, donor nerve transplantation, allografting of various types of nerve grafts like vein allograft, and implantation of Schwann cell in the injury site. The recent treatment strategies included different types of stem cells and nerve conduits.

#### Adipose-derived stem cells (ADSCs)

The ADSCs can be easily harvested from any fat tissue source with no harmful effect and resulted in a high number of cells with very fast culturing and harvesting techniques. It has a good restoration of functional assessment of nerve physiology [34].

#### Bone marrow stem cells (BMSCs)

Mesenchymal stem cells (MSCs), in particular BMSCs, have been demonstrated to be the greatest candidate for regenerating neural tissues. They could rapidly transform into axons and Schwann cells with little immunogenicity and effective immune regulation. They are extremely proliferative and can change into multiple tissue lineages [35].

#### Umbilical cord stem cells (Endothelial progenitor cells)

Schwann cells, brain cells, and axons can all be produced by umbilical cord stem cells (hematopoietic stem cells). Their results are quite encouraging, and positive outcomes have been noted but it needs tissue banks [36].

#### Nerve conduits

A nerve guidance conduit (artificial nerve conduit or artificial nerve graft) is an artificial means of guiding axonal regrowth to facilitate nerve regeneration and it's one of several clinical treatments for nerve injuries. Examples: Collagen nerve conduit, silicone tube, chitosan/fibroin-based nerve scaffold, polycaprolactone nerve conduit (PCL), Silk fibroin -based nerve graft, and polyglycolic acid (PGA) conduit. Using stem cells alone without a guiding material as a nerve graft conduit does not aid in bridging the nerve to re-construct again so most recent research are used these innovative alternatives as a promising guide for cell transplantation [37].

#### Current progress strategies

Recently, miscellaneous types of stem cells and growth factors have been used to produce efficient results in a limited time either alone or with different types of conduits together. Olfactory ensheathing cells (OECs), Autologous dermal fibroblasts, neural stem cells (NCS), Gingival derived mesenchymal stem cells (GMSCs), basic fibroblast growth factor (bFGF), glial cell line-derived neurotrophic factor (GDNF) and gene transfer of adenoviral bone morphogenetic proteins (AdBMP7) have been conducted in recent studies [18, 38–42] (Table 2).

**Table 2:** Illustrates the pros and cons of each type of stem cells

Type of cells	Pros	Cons	References
ESCs	1-*In vitro*-differentiation of various neuronal phenotypes as astrocytes and oligodendrocytes that closely resemble the *in vivo* mature cells.	1-Ethical considerations related to their harvesting and acquisition	[43–48]
	2-Ability to form normal motor neurons, intact axons, and new neuromuscular junction in a degenerated muscle due to nerve injury	2-High incidence of teratoma formation after implantation “tumorigenic cells”	
	3-The newly differentiated cells can produce motor neuron markers and arrest muscle atrophy through cholinergic termination	3-Can be easily rejected from the body as not considered autologous cells and must be injected with immunosuppressive drugs	
NSCs	1-Excellent and good differentiation of neural stem cells into Schwann-like cells that able to bridge gap defects in PNS	1-Difficult to be harvested	[34, 45, 49, 50]
	2-The differentiated cells from neural precursors cells are functional and express motor neuron markers of Acetylcholinesterase and extended axons in the degenerated muscle.	2-The count of cells is not high after harvesting	
		3-Possible damage to the donor nerves used for isolation	
	3-The neurons differentiated from stem cells can produce synaptophysin that enhances neuronal activity	4-Not reliable in cases of trauma of nerves	
BMSCs	1-Easily harvested through bone marrow aspiration with no ethical problems	1-Relatively considered more invasive method for auto-transplantation	[34–36, 48, 51]
	2-Multi-lineage differentiation capacity	2-Cell yield not high enough for implantation after isolation and need for further expansion and culturing	
	3-Secrete neurotrophic factors for regeneration		
	4-The most commonly used in stem cells-based studies		
	5-Low immunogenicity and better expansion for auto-transplantation		
ADSCs	1-Easier isolation and expansion	Not accessible in the skinny body for auto-transplantation	[52–54]
	2-Harvested in more abundance than BMSCs		
	3-Low invasive technique than BMSCs		
	4-Better proliferation and differentiation		
	5-Preferred by authors over others for their easier manipulation and handling		
	6- Fast culturing properties		
HUCSCs	1-Easily obtained from fetal tissues after birth and not ethically limited	Limited availability of fetal sources if not banked earlier	[34, 36, 55]
	2-Good proliferation and differentiation properties		
	3-Decrease the inflammatory response after injury and aid in nerve regeneration		
SKP-SCs	1-Can be differentiated into neural crest precursors cells that have the properties of Schwann-like cells and produce s-100 protein and myelin basic protein that trigger neurogenesis	Technical difficulties related to their slow growth and culturing into Schwan cells “practically no reliable”	[56–58]
HAFSCS	1-Have the properties of both mesenchymal and neural stem cells	Ethical consideration	[48, 59, 60]
	2-Anti-apoptotic and anti-inflammatory activities		
	3-Enhance myelination and axonal growth when injected with hyperbaric oxygen		
MDSPCs	1-Multilineage differentiation	Not broadly used in research basic studies	[56, 61]
	2-Stimulate neuronal regeneration through multiple growth factors secreted from them		
	3-Ability to link between myogenesis and neurogenesis		
	3- High survival capacity under hypoxic and oxidative stress factors		
OECs	1-Have the ability to self-renew into Schwann cells and produce neural growth factors “neurotrophic effect”	Difficult harvest from the olfactory bulb	[8, 34]
	2-Establish channels for newly formed axons and remove dead ones through phagocytosis “neuroprotective effect”		

ESCs “embryonic stem cells”, NSCs “neural stem cells”, BMSCs “bone marrow stem cells”, ADSCs “adipose derived stem cells”, HUCSCs “human umbilical cord stem cells”, SKP-SCs “skin-derived precursor stem cells”, MDSPCs “Muscle-derived stem/progenitor cells”, OECs “Olfactory Ensheathing Cells**”**

### Animal models of PNI regeneration

Most of the candidates in research had resorted to using animal models to help in the modification of the ideal method of treatment to be applied in human therapy strategies in many urgent cases. Rodents are less similar to human immune system, while dogs, cats, swine, sheep, and non-human primates are considered the most volunteers in resembling the human body physiology and ideal to be used to obtain the best evaluation of functional recovery [62].

**Fig. 1:**
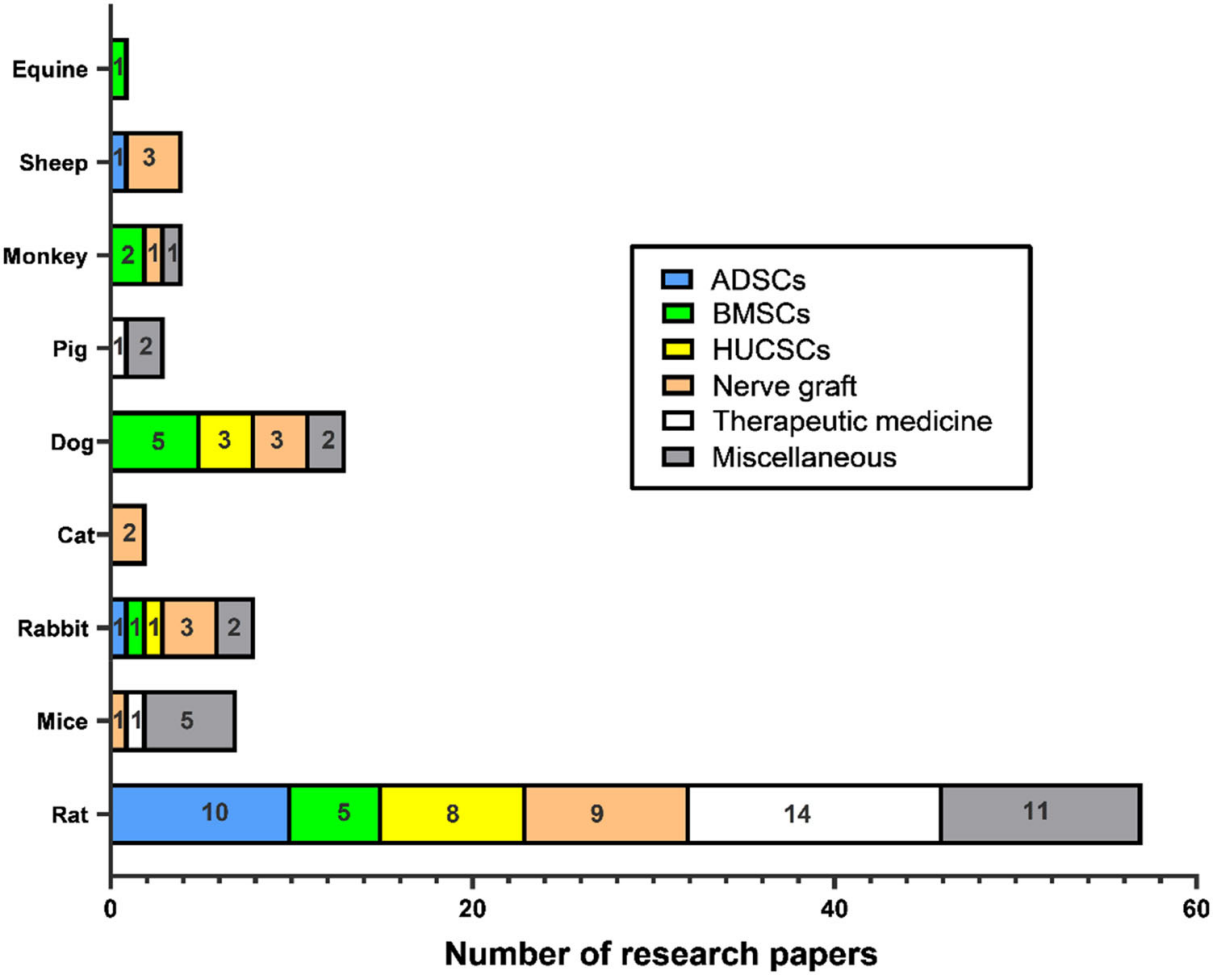
Summary of regenerative strategies involved in PNI

**Fig. 2:**
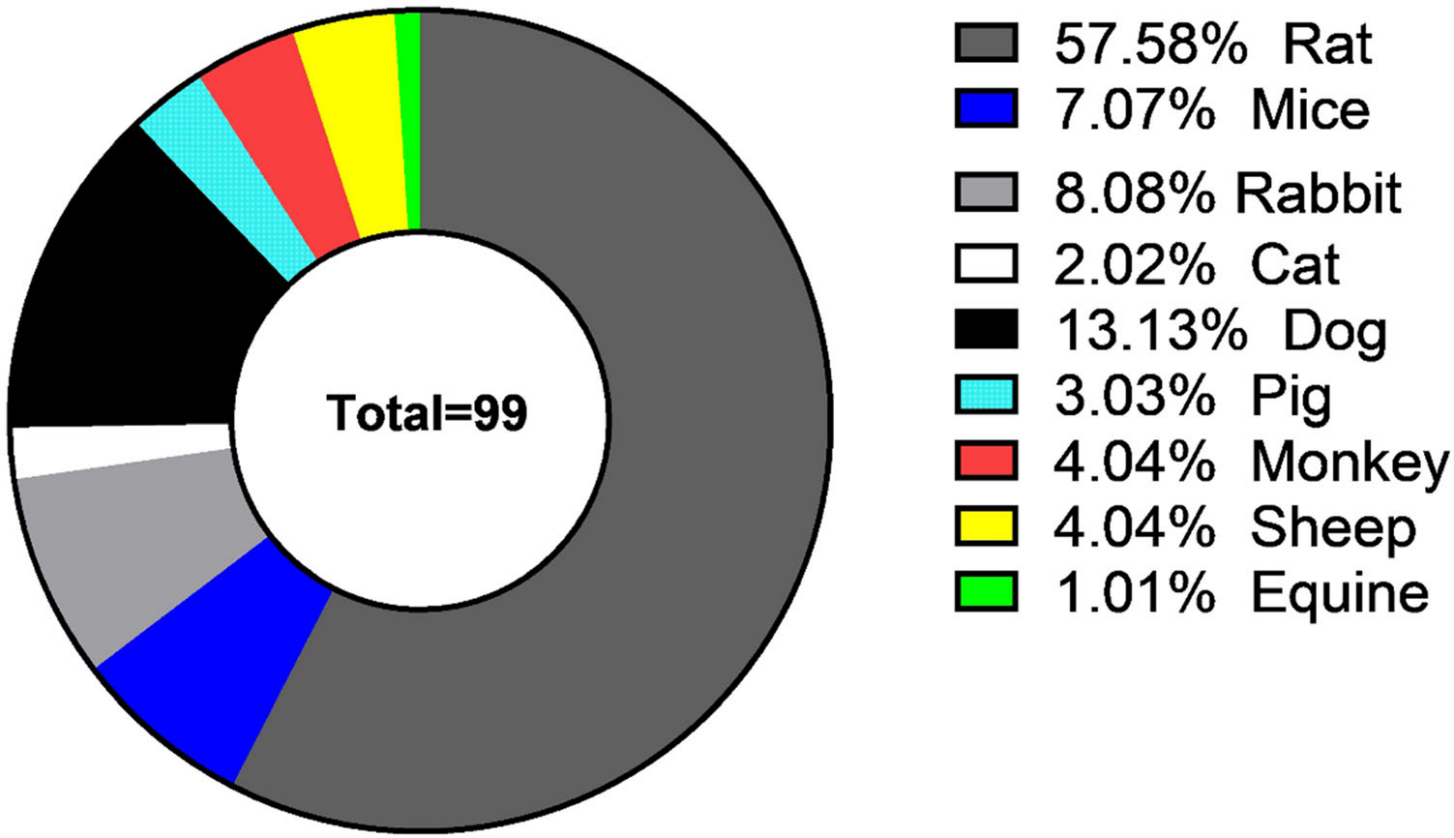
Animal models used in PNI

**Fig. 3:**
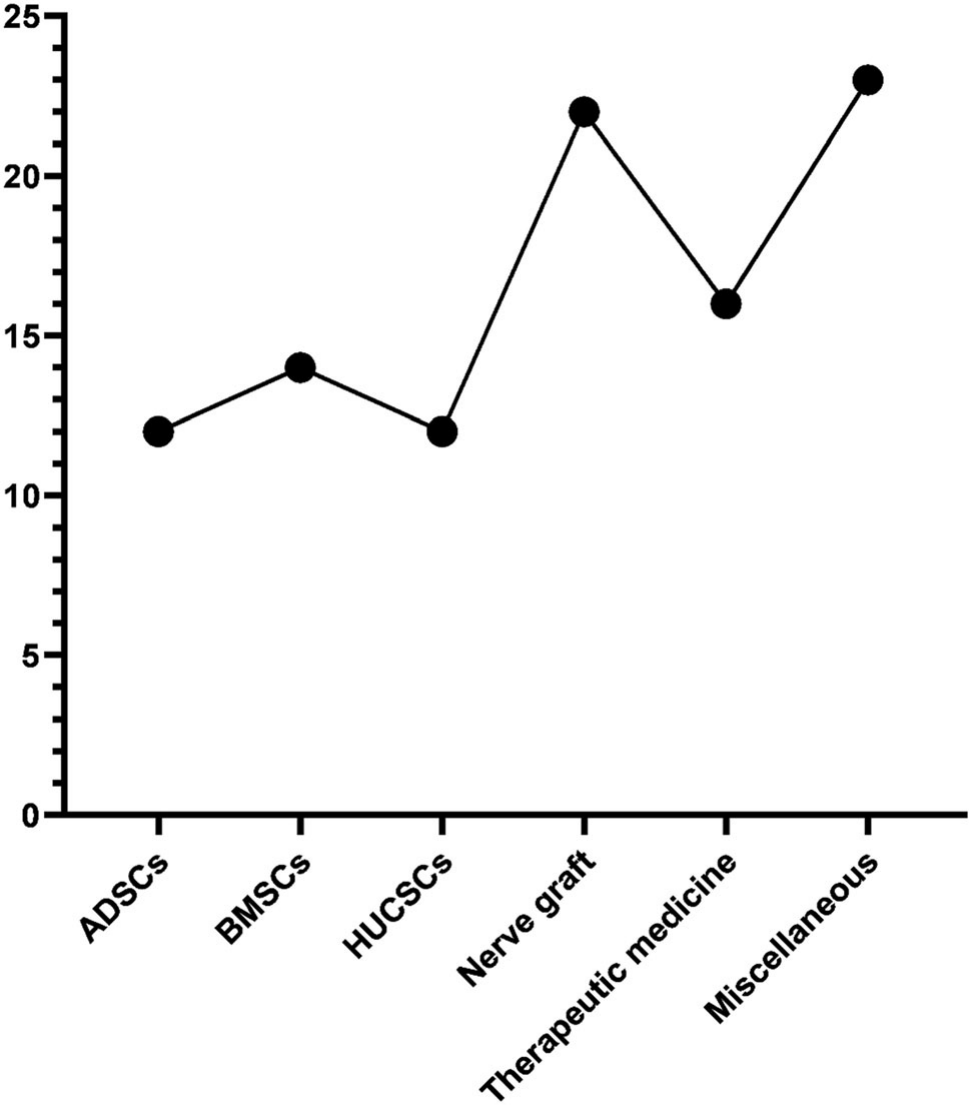
Numbers of trials used stem cells and other therapeutic methods

Concerning experimental studies using rodents as an animal model, we investigate 58 research demonstrate different treatment strategies like using adipose stem cells, bone marrow stem cells, neural crest stem cells, and other types of mesenchymal stem cells also application of various kinds of nerve conduit as ANA conduit, NeuraWrap™, fibrin gel conduit and also administration of many drugs help in improving nerve recovery as alfa-lipoic acid, curcumin, Zofenopril, dexamethasone and methyl cobalamin (MeCbl). Induction of injury is either done by crushing using surgical clump or forceps, or by a transaction of a piece of nerve and filling the gap formed in between the two stumps by conduit material. The follow-up period is differed according to many factors like weight, age, species, and also the methods of evaluation used are confined to behavioral analysis as a sciatic functional index, electrophysiological parameters, histopathological analysis, fluorogold retro-tracing assessment, electron microscope, and gene expression by real-time PCR, Figs. 1, 2 and 3 summarized the different regenerative strategies and animal models used in PNI.

In canine studies, different types of mesenchymal stem cells were used to heal the most affected nerves such as radial in the forelimb and sciatic in the hindlimb. Other methods as autografting of nerve after removal, application of nerve guidance conduit, and injection of nerve growth factors and extra-cellular vesicles are also used. Clinical evaluations were conducted by using histopathology, immunohistochemistry, and functional assessment either motor or sensory. The result was obtained by comparison to control group animals in each parameter and many studies were compared to autografting as considered the ideal technical approach in nerve quality healing.

In feline studies, only two research had reported in the peripheral nerve injury model and approach to treatment by grafting normal body tissue like venules or a synthetic sponge material had proved that nerve recovery after implantation was better unless untreated.

In pigs, rabbits, monkeys, and sheep, most of the research was found helpful in manipulation of nerve injury by using stem cell therapy together with nerve grafting after several types of nerve injuries. So, nowadays after all of these articles, there is no difficult way to apply any of these modern approaches when facing different types of nerve injuries by backing and following up the newest published articles.

### Effect of animal models in the direction of research study in PNI

Regarding the animal that can be selected according to the effect of different therapeutic methods on an injured nerve, each animal has its point of selection. Murine species (rats and mice) are the most frequently used on a small animal scale, they are considered more economic, easily handled, housed, and investigated in large groups that became more accurate and representative, although, the gap applied in this species (almost not exceed 10 mm) which is relatively shorter than those of a human gap, also suspected time for complete healing is very short compared to human nerve injuries. ultrasonography required for following up the improvement cascade was almost difficult to be applied besides the genomic material of this species being lower than that of human beings [63, 64].

Canine and feline species are considered heavily weighted animals. Their weight helped in typical clinical signs of peripheral nerve injuries such as knuckling syndrome in sciatic neuropathy, so facilitate follow-up of the case and the degree of improvement. In addition, this species is relatively more similar to human genomic basis and produces more representative outcomes after a certain period of treatment. On the other hand, this species has an ethical problem with its use in research studies, they require high cost for purchase, housing, and care during the experimental period and the availability is limited in certain countries [65, 66].

Rabbits are one of the most commonly used animal models in nerve regeneration studies and their gaps are near to those that occur in humans, but they have a high cost of purchasing and housing, also they need an intense care system. Rabbits’ life is short, they rarely exceed five months, so this period is not enough for large gap studies.large animal models like pigs, sheep, equine, and non-human primates are limited in experimental studies due to their very high-cost, difficult housing, care, and, evaluation of the study, However, the absolute similarity between human and non-human primates became a promising challenge for testing the appropriate methods and efficacy of treatment strategies before applied in human being.[67–69].

The choice of an appropriate animal model in future research studies depends on the direction of research goals and ideas. Rats and mice will remain the most commonly used models in research studies for their advantages mentioned above, However, human injury defects can be representative by large models such as dogs and monkeys that are limited as their high-cost issues and ethical concerns. So, according to the aims of the designed study and the outcome expected to be obtained, the authors are directed to their best choice of the animal model.

### Some clinical trials reported in pni using stem cell therapy

Although several research studies investigate the stem cell protocol in the treatment of animal models undergoing peripheral nerve damage, however in most vet clinic cases using these technologies remains almost rare. But there were various cases documented in human using these trials. A study was conducted by the University of Miami, Florida, United States, they used autologous human Schwan cells augmentation in several nerve injuries (ClinicalTrials.gov Identifier: NCT05541250). Another study was presented by Zhang Peixun, Department of Orthopedics and Trauma, Peking University People's Hospital, they investigate the Mid-term clinical effect of biodegradable conduit small gap tubulation to repair peripheral nerve injury in multi-center (ClinicalTrials.gov Identifier: NCT03359330).

#### Future prospectives of stem cell therapy in PNI

Several studies have proved that stem cell-based therapy has the potential to induce nerve regeneration and axonal remyelination, but they differ in their preference for which type performs the greatest and the highest efficacy. Seyed-Forootan et al., 2019 showed that ADSCs and BMSCs have the greatest power of neural regeneration among other types of stem cells and added that BMSCs are the best choice in this filed [34]. On the other hand, Dadon-Nachum et al., 2011 recommended that Stem cells obtained from bone marrow, adipose tissue, amniotic fluid, and hair follicles are the most commonly used types. Recently stem cells were used with growth factors such as PRP, glial cell line-derived neurotrophic factor, and basic fibroblast growth factor, more over medicine nowadays was directed to the use of extracellular vesicles of stem cells (exosomes) that help in providing a favorable microenvironment for peripheral nerve regeneration via mediating axonal growth and regulate inflammatory cascade after injury. Overall, we believed that stem cell therapy became an accessible routine in the treatment strategy of different kinds of PNI and is considered the better regenerative medicine for the improvement of traditional old therapeutic interventions.

## Conclusion

Peripheral nerve injuries had a limited regeneration capacity particularly when the nerve gap exceeds the possible degree of extend, the nerve axon couldn’t be able to reconstruct a new nerve tissue so maintenance of the Peripheral nerve's proper functionality after injury becomes an intractable challenge for clinical researchers. Nowadays, most of the clinical trials involved in treatment of this PNI are directed to different types of mesenchymal stem cell therapies as an alternative to nerve neurorrhaphy to obtain high-quality healed nerve tissue. In our review, we conclude that nerve grafting applications together with stem cells alone or with other growth factors have the best scoring of nerve repairing capacity. After that, the use of nerve conduit alone came later to help Schwan cell in building of new axons and enhance the reverse of Wellerian degeneration. However, other techniques like systemic injection of therapeutic medicine such as nerve tonics and natural antioxidants gave good results in much research and opened a way to interpose in this innovated medicine (Tables 3–8).

**Table 3:** Illustrate adipose derived stem cells therapy research

Animal	Nerve involved	Type of therapy	Type of injury	Injected dose	Type of conduit	Clinical evaluation	Clinical results	References
Rat	Sciatic nerve	Adipose-derived stem cell	Removal of about 8 mm of nerve remaining 10 mm gap	Not recorded	ANA conduit (15 mm in length)	Functional assessment Electrophysiology, Muscle wt., Histological assessment, RNA isolation & Tissue immune fluorescence	1-SFI improved after 12 weeks.	[8]
							2-Electroneurograph show improved velocity, latency, and nerve amplitude	
							3-Increase in number and diameter of nerve fibers &myelin sheath.	
Rat	Sciatic nerve	Adipose derived stem cells	10 mm portion of the nerve is removed	1 × 10^5^ neurally differentiated cells in a fibrin glue	A silicone tube	Nerve Conduction Study, Histopathologic Examinations, Immuno-cytochemistry Examinations and Electron Microscopic Examination	1-The NC group expressed more nestin, S100, and GFAP than other groups	[70]
							2-One-way ANOVA showed that the mean amplitude of affected sides was greatest in the NC group (20.25 ± 5.23 mV) followed by the ADSCs group (17.93 ± 4.19 mV), control group (4.90 ± 2.75 mV)	
Rat	Sciatic nerve	Adipose derived stem cells	Removal of 10 mm	10000 ADMSCs	Contain Collagen nerve conduit	Electrophysiological evaluation, Clinical assessment, histological assessment, and immunohistochemistry	1-after 12 weeks, higher re-myelination in the C-FAH group than others.	[71]
							2-S100 and laminin protein expression displayed a regular, organized pattern and were noticeably more intense and abundant in the C-FAH group	
Rat	Sciatic nerve	Adipose-derived stem cells	Removal of about 15 mm long segment	4 × 10^6^ cells/mL of gel	NeuraWrap™ sheath (18 mm long)	Immuno-fluorescence imaging and transmission electron microscopy	1-Immuno-histochemistry in multiple locations within the repaired nerve tissue PS, PD, DD, DS) show that EngNT-ADSCs conduits supported 3.5-fold more regenerating axons than the empty tube Controls in both the DD and DS	[19]
Rat	Sciatic nerve	Differentiated adipose-derived stem cells (ADSCs) + Primary Schwann cells (SCs)	Removal of 10 mm of nerve	80 × 10^6^ cells/mL	Prepared poly-3-hydroxybutyrate strips (PHB)	Walking track analysis, EMGs, muscle weight ratios, and muscle and nerve histology	1-The nerve conduction velocities were highest in the ADSCs and SC groups	[72]
							2-In both treated groups there were a significantly higher total number of myelinated axons in the distal stumps than control group.	
							3-In the animals treated with either type of cell, there was significantly less atrophy of muscles than in the control	
Rat	Sciatic nerve	Adipose-derived stem cell	20 mm long segment was removed	3 × 10^6^ cells	Fibrin gel conduit of a length of 25 mm	SFI and Histological evaluation	1-In the first 4 weeks after surgery no difference between experimental groups in SFI.	[73]
							2-At 16-week, animal show improvement and increase axon density and myelination, Increase G-ratio	
Rat	Sciatic nerve	Adipose derived stem cell + Schwann cell	Removal of 8 mm of nerve with a 10 mm gap	1 × 10^6^ cells/mL in 25 μL suspensions of ADSCs	A tissue-engineered nerve (ANA)	Walking track analysis, Electrophysiological analysis, Histological analysis, Immunofluorescence staining, Western blot analysis, and Real-time PCR analysis	1-ANAs in combination with Schwann-like cells inhibited JAK2/STAT3 signaling pathway activation through increasing expression of nerve fibers in spinal cord	[74]
							2-In the SC-L group, the NCV and WA increased (P < 0.01), twelve weeks after surgery	
Rat	Sciatic Nerve	Adipose derived stem cell	Crushing of nerve for 60 seconds.	A million cells were implanted by perineural injection	Without conduit	SFI, muscle mass measurements on tibial and gastrocnemius muscles	1-Significant difference observed in SFI after 2,4weeks	[1]
							2-Significant difference in amplitude by electromyography after 4 weeks but no difference in latency	
							3-Increase in weight of muscles after 3 weeks of surgery	
Rat	Sciatic Nerve	Adipose-derived stem cell	20 mm transaction of nerve	1 × 10^6^ cells of ADSCs	silicone tube	Behavioral assessment, Foot fault score, and Histological evaluation	1-At 3-month scores of foot fault increased by 59% compared to the first month.	[4]
							2-3 months post-transplantation, sections show axon regeneration and re-myelination	
Rat	Sciatic nerve	Adipose-derived mesenchymal stem cell (ADSCs) SPIONs-treated	Crushing of nerve for 8 s	6–7 × 10^6^ ADSCs-SPIONs-treated injected in the lateral tail vein	Without conduit	Epifluorescence, confocal, transmission electron microscopy, Immunofluorescence, Electrophysiological measurements and Image analysis	1-ADSCs/MT systemically transplanted as early as the 7th day post-surgery accelerated the improvement in MBP organization encouraged by ADSCs.	[75]
							2-Remyelination was significantly enhanced by ADSCs/MT, with a mean difference of 41.2 % compared to other groups	
Rabbit	Peroneal Nerve	Human adipose derived stem cell	A segment of the nerve was removed with a 40 mm gap	1 × 10^6^ stem cells	autologous gluteal vein graft conduit	Macroscopic study, Microscopy findings (Hematoxylin-eosin & Electron microscopy) and Neurofilament immunohistochemistry “mouse antihuman neurofilaments” (anti-NF, 2F11)	1-At 21 days the circumference of regenerated nerves was equal to the peroneal nerves	[76]
							2-Nerve regeneration throughout the vein was confirmed without evidence of inflammatory activity even with human ADSCs	
							3-Treated group contained more myelinated axons per field (5.2 _ 2.0 per field)	
							4-Cells exhibited a positive reaction at the cytoplasm	
Sheep	Sciatic nerve	Adipose derived stem cells	20 mm long segment was removed	3 × 10^5^ Schwann-like cells differentiated from ADSCs	xenograft and autograft	Neuro-physiologic evaluation, Evaluation of muscular atrophy & morphological evaluation	6 months after surgery,	[77]
							1-Myelinated neurofilaments and s-100 protein were observed in both AGT & xenograft groups.	
							2-no significance difference in amplitude of gastrocnemius & biceps femoris muscle in both AGT & xenograft groups	

ANA “A cellular nerve graft”, NC “neurally differentiated cells”, GFAP “Glial fibrillary acidic protein”, ADSCs “adipose derived stem cells”, C-FAH “collagen fibrin agarose hydrogel”, PS “proximal stump”, PD “proximal part of device”, DD “distal stump”, DS “distal part of device”, EngNT-ADSCs “engineered nerve tissue - adipose-derived stem cells”, SFI “sciatic functional index”, G-ratio “the ratio of the inner-to-outer diameter of a myelinated axon”, JAK2/STAT3 “Janus kinase 2/signal transducer and activator of transcription 3”, SC-L “Schwann cell-like”, NCV “nerve conduction velocity”, WA “wave amplitude”, SPIONs “superparamagnetic iron oxide nanoparticles”, ADSCs/MT “adipose derived stem cells/magnetic targeting”, MBP “myelin basic protein”, AGT “autograft”.

**Table 4:** Illustrate bone marrow derived stem cells therapy research

Animal	Nerve involved	Type of therapy	Type of injury	Injected dose	Type of conduit	Clinical evaluation	Clinical results	References
Rat	Sciatic nerve	Bone marrow stromal cells (BMSCs)	Transection of 5 mm	50, 000 BMSCs in 5 mL of culture medium injected into the distal stump of the nerve	Without conduit	Functional assessment of sciatic function index (SFI) and Dual immune-fluorescence labeling	1-In compared to the control group, BMSCs enhance functional recovery by at least 36% and 78% after 18- and 33-days respectively after nerve transection	[78]
							2-At day 33, a complete and uniform connection between proximal and distal stumps in nerves	
Rats (male Sprague–Dawley)	Sciatic nerve	Bone marrow stromal cells (BMSCs)	Removal of 15 mm	1×10^6^ BMSCs cells/tube suspended with 2% gelatin	A 20 mm silicone tube	Functional assessment, Electrophysiological study, Histological examination and (RT-PCR)	1-10 weeks after surgery, the percentage of activated fibers was higher in the BMSCs-implanted group than in the control group.	[79]
							2-A higher level of neurotrophic factor expression was accompanied by BMSCs group incorporated in regenerating tissues	
Rat	Sciatic nerve	Bone marrow mesenchymal stem cells	Crushing of nerve for 30 s	1.5×10^6^ BMSCs in 1 mL of PBS injected in the tail vein	Without conduit	Light and electron microscopic studies, Morphometric and statistical study	Subgroup IIIa "sacrificed after 1 week" & subgroup IIIb " sacrificed after 2 weeks " showed the same histological parameters " proliferation of Schwann cells columns invaded by sprouts of regenerating sciatic nerve fibers"	[80]
Rat	Sciatic nerve	Bone marrow mononuclear cells (BM-MNCs)	Removal of a 10 mm of nerve	1×10^8^ cells/mL of BM-MNCs	A chitosan/fibroin-based nerve scaffold	Gait analysis, Histological Observation, Morphometric Analysis, electrophysiological, Immunohistochemistry assessment and Transmission Electron Microscopy	1-At 12 weeks better metatarsophalangeal joint plantar flexion and toe spreading	[81]
							2-axonal re-growth gradually increased with time	
							3-motor nerve conduction velocity increased in BM-MNC & autograft group only	
Rat	Sciatic nerve	Bone marrow derived (BMSCs) + Granulocyte-Colony Stimulating Factor (G-CSF) and/or Dihexa growth factor	Transaction of the sciatic nerve	2 × 10^6^ BMSCs injected locally and 1.0–1.5 mL I/V into the dorsal penile vein	Without conduit	Sensory function analysis, Motor function analysis Gastrocnemius muscle mass wt., Foot flexion contractures assessment.	1-At 16 weeks after surgery, none of the treatment groups show a significant reduction in the loss of gastrocnemius muscle mass.	[82]
							2-When compared to the control group 10, flexion contracture decreased in group 7 (Grade 1.2), which received G-CSF + BMSCs, and group 8 (Grade 1.8) which received Dihexa + BMSCs.	
Rabbit	Brachial plexus (C5-T1)	Bone marrow mesenchymal stem cell	Expose roots of C5-T1 (ventral & dorsal) then torn off from the spinal cord	5×10^6^ RFP-BMSCs were injected intraperitoneally	Without conduit	The recovery rate of the wet weight of the upper limb muscle, hematoxylin and eosin (HE)staining of injured tissue and electrophysiological measurement	3 weeks from surgery: 1-increased recovery rate of wet muscle weight (*p*<0.05)	[83]
							2-The number of axons, myelinated fibers, and the density of nerve was raised	
							3-The levels of p-MAPK and p-ERK were lowered	
							4-The CMAP amplitude was reduced markedly (*p*<0.05).	
Dog	Sciatic nerve	Autologous- bone marrow mesenchymal stem cells (BMSCs)	50 mm long removed from the nerve	8 × 10^7^ cells	Chitosan/PLGA-based neural scaffold	Electrophysiological assessment, Fluorogold retrograde tracing & Histological assessment.	1-Six months after grafting dogs in three grafting groups were able to stand up straight on two hind limbs	[84]
							2-No significant difference in CMAP between 3 grafted groups	
							3-Between the three groups (scaffold > TENG > autograft), the thickness of the regenerated myelin sheath showed a progressive increase	
Dog	Sciatic nerve	Autologous bone marrow mesenchymal stem cells (BMSCs)	60 mm long is removed from the nerve	1 × 10^8^ mL BMSCs	Chitosan with poly lactic-co-glycolic acid (PLGA)	Behavioral Analysis Electrophysiological Assessment, Fluorogold (FG) Retrograde Tracing & Morphometric Analyses.	1-Massive bundles of myelinated nerve fibers that were double-labeled by NF and S-100 were found in the TENG group at 12 months after surgery.	[20]
							2-Between the autograft and TENG groups in CMAP, there was no discernible difference.	
Dog	Sciatic nerve	Bone marrow mesenchymal stem cells- a cellular matrix (BMSC-ACM)	45 mm long segment removed leaving a 60 mm gap	1 × 10^6^ cells	Chitosan/silk fibroin-based neural scaffold	Behavioral analysis, Electro physiological assessment& Fluro Gold retrograde tracing, Histological assessment and Morphometric analysis	1-12 months post-surgery, animals showed better stability of stance and greater restoration of locomotive activities.	[21]
							2-A recovery ratio of (CMAP & MCV) was about 50% at 12 weeks post-surgery	
Dog	Sciatic nerve	Bone marrow mesenchymal stem cells (BMSCs)	20 mm segment removed with 30 mm gap	Not recorded	Silk fibroin -based nerve graft conduit (NGC)	Behavioral analysis, Electrophysiological, tests and Fluorogold (FG) retrograde tracing, Histological assessments and Morphometric analysis	1-The majority of the SF-based nerve graft was replaced at 12 months after surgery by tissue that looked like nerve tissue.	[85]
							2-After 12 months, No significance difference between autographed & scaffold groups in CAMP and % of FG-labeled motor & sensory neurons.	
Dog	Sciatic nerve	Bone marrow mesenchymal stem cells (BMSCs) + injection of testosterone propionate (TP)	30 mm long segment was removed	1 × 10^7^ cells per mL	A cellular nerve allograft (ANA)	Electrophysiological assessment, Fluorogold retrograde tracing & Histological evaluation	Five months postoperatively,	[22]
							1-CMAPs in the TP+ANA+BMSCs group were higher than that in the ANA+BMSCs group	
							2-In the TP+ANA+BMSCs group, both the diameter ratio of the myelinated nerve and the thickness ratio of regenerated myelin sheath were significantly larger than in the other groups	
Rhesus Monkey	Radial nerve	Autologous bone marrow stem cells	10 mm removed from the nerve	2 × 10^6^ BMSCs	A cellular allogeneic nerve conduit (ANA)	Electro- physiological analysis, immuno-fluorescence staining & histomorphometric analysis	1-Sections from the distal end of the allogeneic graft show a high density of well-myelinated fibers in comparison to the control group.	[86]
							2-At 8-week post-surgery, the allogeneic grafted group seeded with MSC show higher NCV, amplitude & shorter latency of CMAP than groups grafted without cells.	
Rhesus monkey	Median nerve	Autologous- bone marrow stem cells(BMSCs)	50 mm long nerve gap.	1 × 10^8^ mL of cells	Chitosan/PLGA scaffold	Behavioral and safety assessment, Electrophysiological assessment and fluorogold retrograde tracing and histopathological assessment.	1-At 12 months hand motility was better in the autograft while no change was in the non-graft group.	[87]
							2-Between the three grafted groups, there were no perceivable differences in the proximal CAMP amplitude and MCV.	
							3-The scaffold group had a lower % of FG-labeled sensory neurons in DRGs	
							4-The density of NF-positive fibers was significantly higher in the TENG group	
Horse	Ramus Communicans	Horse bone marrow stem cells (BMSCs)	A 15 mm long incision	10 × 10^6^ cells in 1 mL of sterile isotonic saline injected into the fascia surrounding the nerve	Without conduit	Behavioral evaluation, histological evaluation of BMSCs into SLCS and immunohistochemistry of SLCS protein expression.	1-No horses exhibit any form of disability after the operation.	[88]
							2-SLCS from EBW-MSCS success in the expression of S-100b and GFAP but undifferentiated control cells express β_3_ tubulin and GFAP and failed to express S-100b “neural progenitor markers”.	

RFP-BMSCs “red fluorescent protein-bone marrow stem cells”, p-MAPK “protein- mitogen-activated protein kinase”, p-ERK “protein-extracellular signal-regulated kinases”, CMAP “compound muscle action potential”, TENG “tissue engineered nerve graft”, NF “neurofilament”, MCV “muscle conduction velocity”, SF-based “silk fibroin-based”, FG-labeled “fluorogold-labeled”, DRGs “dorsal root ganglions”, SLCS from EBW-MSCS “Schwann-like cells from equine bone marrow-mesenchymal stem cells”, GFAP “Glial fibrillary acidic protein”, β_3_ tubulin “class 3 tubulin protein”.

**Table 5:** Illustrate human umbilical cord stem cells therapy research

Animal	Nerve involved	Type of therapy	Type of injury	Injected dose	Type of conduit	Clinical evaluation	Clinical results	References
Rat	Sciatic nerve	Human umbilical cord derived mesenchymal stromal cells (HUCSCs)	An 8 mm was completely removed	1-2 × 10^7^ cells/mL suspension	Artificial graft (8 mm long) trans permeable tubes (hollow fibers, Amicon, Beverly, MA)	Immunohistochemistry, Immunoelectron Microscopy, and Walking Track Analysis	1-The UC-SC group exhibited significantly better functional improvement than other experimental groups at 21 days post-surgery 2-Many cells with gold particle-labeled cytoplasm were seen closely linked to newly growing axons and myelin sheaths in the UC-SC group	[23]
Rat	Sciatic nerve	Human umbilical cord matrix MSCs (HUCSCs)	Crushing of 3mm for 30 s	Suspension of 1500 HUCSCs (In a total volume of 50 mL)	Poly (DL-lactide-e-caprolactone) (PLC) membranes	Sciatic functional index (SFI), extensor postural thrust (EPT), and withdrawal reflex latency (WRL)	1-Compared to the group treated with PLC membrane alone, the groups treated with cells plus PLC membrane displayed great variability in EPT values. 2-SFI values in treated groups gradually increased until, by week 12 of recovery, they were comparable to those of control animals.	[89]
Rat	Sciatic nerve	Non-differentiated human mesenchymal stem cells isolated from Wharton’s jelly of umbilical cord	Crushing of 3 mm of nerve for 30 seconds	A suspension of 50 μL (1 250–1 500 HUCSCs)	Chitosan type III	Functional analysis of motor deficit (Sciatic functional index, static sciatic index, extensor postural thrust, and withdrawal reflex latency) Sciatic nerve morphology and stereology	1-At week 12 post-surgery, the WRL improved in all animals 2-Among different experimental groups, the Crush chitosan III group had significantly (*P* < 0.05) lower fiber density and fiber total number and a higher myelin thickness	[24]
Rat	Sciatic nerve	Human umbilical cord blood-derived mesenchymal stem cells	Crushing of nerve for 1 minute	1 × 10^6^ cells/15 μL of PBS injected into the crushed nerve	Without conduit	BDNF, TrkB, and p75 mRNA expression detection, Gait analysis with SFI, Retrograde labeling, and Histomorphometric analysis	1-BDNF and TrkB mRNA expression was significantly higher in the cell group than control starting from Five days after the injection “increased by 2.48”. 2-Fluro Gold-labeled neuron counts in the cell group was 118.96 ± 12.13 which was higher than the control group	[90]
Rat	Sciatic nerve	Human umbilical cord mesenchymal stem cell	Removal of about 6-8mm segment of nerve	1 × 10^5^/mL	Amnion tube isolated from the internal surface of the fetal membrane	Sciatic functional index, electrophysiological indices, wet weight of gastrocnemius Muscle and Histopathological analysis	1-At weeks 8, 12, 16, and 20, the cell transplantation group's wet weight and restoration ratio of gastrocnemius Muscle were higher than those of the control group. 2-Threshold stimulus and maximum stimulus intensity differed in the cell transplantation group than control (*P* < 0.01)	[18]
Rat	Femoral nerve	Human mesenchymal stem cells-umbilical cord blood (HUCSCs)	streptozotocin-induced diabetes causing femoral neuropathy was injected intraperitoneally	2.0 × 10^6^ HUCSCs/rat were administrated through the left femoral artery	Without conduit	Serum NGF examined by ELISA, light microscope, immunohistochemistry, transmission electron microscopy, and Electroneurogram (ENG)	1-Serum NGF level at 3rd day after treatment with HUCSCs was partially increased to 93.1 ± 15.7 ng/l. 2-NF-200 immunostaining was dramatically enhanced in treated models at 14d after HUCSCs treatment.	[91]
Rat	Sciatic nerve	Human umbilical cord blood-derived mesenchymal stem cells (HUCSCs) + BDNF-Ad “adenovirus vector-mediated brain-derived neurotrophic factor”	Crushing of the nerve use of a standard hemostat	1 × 10^6^cells/10μl injected into the crushed nerve	Without conduit	Functional assessment via Sciatic Functional Index (SFI), Retrograde axonal transport, and histomorphometric analysis	1-At the end of the 3rd and 4th week, the BDNF-Ad+ HUCSCs group exhibit a higher average recovery level of SFI than the control group. 2-4 weeks post-surgery, the BDNF-Ad+ HUCSCs group showed significantly higher axon density compared to the control group (*p*=0.034).	[92]
Rat	Sciatic nerve	Human umbilical cord MSC‐derived extracellular vesicles (HUCSCs‐EVs)	Removal of 3mm of nerve with a 5 mm gap	100 μg of HUCSCs ‐EVs (100 μL) in 0.2 mL of PBS was injected I/V into the tail vein	Without conduit	Functional assessment, Muscle weight measurement, Immunofluorescence, Transmission electron microscope, and Hematoxylin and eosin staining	1-The SFI scores for the HUCSCs ‐EV group increased at 4, 6, and 8 weeks after surgery 2-The diameters of the regenerated nerve fibers in the HUCSCs -EV group were higher than the control at 8 weeks after injury of the nerve 3-Axonal regeneration exhibited by the HUCSCs ‐EV was better than the control	[93]
Rabbit	Tibial nerve	Human umbilical-cord-derived mesenchymal stem cells (HUCSCs)	Removal of 10 mm from nerve	0.1–0.025 mL cell suspension	Chitosan nerve conduit “An inner diameter of 2 mm, wall thickness of 0.5 mm, and length of about 10 mm”	Morphological Evaluation, Anti-S-100 Immunohistochemistry and Electrophysiological Examinations of Regenerated Nerves	1-Nerve conduction velocity in group C (control group) was greater than that in group A or group B (treated group). 2-The number of medullated fibers and the myelin sheath thickness in group C were higher than those in group A or B. 3-A large number of brown-red proliferating Schwann cells was found in group C but not in others	[94]
Dog	Sciatic	Human umbilical cord mesenchymal stem cells (HUCSCs)	35 mm of the nerve was removed	1 × 10^6^ HUCSCs	(LOCC) “longitudinally oriented collagen conduit”	Electrophysiological measurements, Electron microscopy, Histological analyses & Muscle mass analyses	1-9 months after surgery, nerve conduction was better in the LOCC/HUCSCs group than in the LOCC-alone group. 2-In the middle of the regenerated segment in the LOCC/ HUCSCs group, GAP-43-positive, NF-positive, and S-100-positive cells were found 9 months after surgery.	[95]
Dog	Radial nerve	HUCSCs	Removal of 1 cm of nerve	Not recorded	Without conduit	Clinical& physical examination, Immunological& hematological Evaluation	1-IgG…. Increased at. 4,6th week & then decrease at 8,16th weeks. 2-ILS high in 2,4th weeks & then low in 6, 8,16th weeks. 3-WBC high in & 4, 6th weeks then low in 6, 8, 16th weeks. 4-2–5th week's slight extension of the forelimb. 5-16th week return to normal with no lameness.	[6]
Dog	Radial n	A cellular lyophilized Human umbilical cord extracellular matrix (HUCSCs HUC-ECM)	1 cm is removed	0.01 mg of Acellular lyophilized HUCSCs -ECM	14 mm A cellular bovine urinary bladder matrix (UBM) conduit	Neurohistopathological assessment	1-At 56th day post-surgery, signs of regeneration start to appear in the treated group than in the control group. 2-At 112th day post-surgery in the treated group, good re-myelination, and higher no. of Schwan cells.	[96]

UC-SC group “umbilical cord-Schwann cell group”, EPT values “extensor postural thrust”, p75 “neurotrophin receptor”, BDNF “brain-derived neurotrophic factor”, TrkB “tyrosine kinase receptor B”, GAP-43-positive “Growth Associated Protein 43**”,** NF-positive “Neurofilament Protein”, IgG “Immunoglobulin G”, ILS “interleukins”

**Table 6:** Illustrate miscellaneous types of stem cells and growth factors therapy research

Animal	Nerve involved	Type of therapy	Type of injury	Injected dose	Type of conduit	Clinical evaluation	Clinical results	References
Rat	Sciatic nerve	Human amniotic mesenchymal stem cells	Transaction of 5 mm of nerve	A volume of 50 microns of MSCs with a density of 10^5^ cells/mL	fibrin glue and woven oxidized regenerated cellulose gauze (Surgical; Ethicon, Somerville, NJ)	Ankle kinematics and sciatic function index (SFI) after 8 weeks of surgery, electrophysiological and immune histochemistry.	1-The average angle of the ankle in the treatment group was 46.4%. 2-Histologically 70% of animals achieved a maximum axon diameter % across the nerve gap.	[97]
Rat	Sciatic nerve	primary Schwann cells + Adult stem cells “Adipose and bone marrow stem cells” differentiated to a Schwann cell-like phenotype	Transaction of 10mm long nerve	2 × 10^6^ cells suspended in 50 μL of differentiation medium	Fibrin nerve conduit “14 mm in length, with a 2-mm lumen and 1 mm wall thickness”	Immunohistochemistry	The distances of the S100 positive cell pattern were: SC group (5.858_ 0.13 mm) followed by a conduit containing BMSCs (5.035_ 0.21 mm) or ADSCs (5.032_ 0.20 mm) 2 The best regeneration distance was demonstrated by conduits filled with SCs (5.758 0.12 mm), followed by fibrin-containing BMSCs (4.986 0.19 mm) or ADSCs (4.968 0.22 mm).	[54]
Rat	Sciatic nerve	Gene transfer of adenoviral Bone morphogenetic proteins (AdBMP7) + adenoviral green fluorescent protein (AdGFP)	Transection 5 mm distal to the sciatic notch + crushing for 10 s	2 μL of AdGFP or AdBMP7 containing a titer of 10^6^ pfu was injected into the nerve	Without conduit	Evaluation of functional recovery, immunohistochemistry and RT-PCR, and nested PCR	1-At days 4 and 7 after injury, AdBMP7-treated rats showed early signs of sciatic nerve Recovery while at 28 days show greater SFI improvement than AdGFP infected group Rats 2-5th weeks after crush showed more intact and regenerated axons in AdBMP7-transducted rats than that in AdGFP transduced rats	[98]
Rats	Sciatic nerve	Glial cell line-derived neurotrophic factor (GDNF)	A 5 mm segment In the middle of the nerve was excised “neurotmesis”	0.1 mg/mL of GDNF loaded on Keratin gel of human Hair	Polycaprolactone nerve conduit (PCL)	Histology and Gastrocnemius Muscle Harvest, Gastrocnemius Muscle Weight Ratio, and Immunohistochemistry	1-At 6 weeks, there is no significant difference in gastrocnemius muscle weight loss ratio between saline (74.17 _ 1.32) and keratin (74.78 _ 2.77) treatment. 2-S100 antibody-stained nerves revealed more Schwann cell-specific S100 protein density in the keratin gel-filled group than control (*p* 0.05).	[99]
Rat	Sciatic nerve	Olfactory ensheathing cells (OECs)	Removal of 15 mm long segment from nerve	3 × 10^6^ cells of OECS suspension	Poly (lactic-co-glycolic acid) “PLGA” conduit	Electro-physiological evaluation, Histological evaluation and Immunohistochemical analysis.	6 weeks postoperative data showed that: 1-higher values of NCV compared to those observed at 2-and 4 weeks post-surgery 2- presence of Schwann cell-like cells that were actively secreting extracellular matrix by electron microscope	[39]
Rat	Sciatic nerve	Neural stem cells (NSCs) + fibroblast growth factor 1 (FGF1)	15 mm was excised	10^5^ cells and 4 μg /mL of FGF1	PLA conduits (Tube A) and PLA conduits containing bioactive components (Tubes B–D)	Functional assessment (walking analysis), Electro-physiological evaluation and Histological analysis	1-The capacity for regeneration of various combinations ranked as autograft > Tube D+ NSC > Tube D > Tube B+ NSC > Tube B > Tube A+ NSC > Tube C > Tube A at six weeks. 2-From 1 to 12 weeks, all groups' SFI and the left-to-right footprint area ratio increased 3- At 12 weeks, groups receiving tube D + NSC or Tube D had a higher SFI than the other groups.	[100]
Rat	Sciatic nerve	Bone marrow stromal cells (BMSCs) + human umbilical cord stromal cells (HUCSCs)	Removal of 10 mm of nerve	500,000 cells	A 12 mm silicone tube	Immunohistochemistry, Light microscopy, Electron microscopy, and Histomorphology of the muscle	1-A newly formed perineurium containing nerve fascicles of myelinated and unmyelinated axons together 2-at 12th weeks neovascularization was observed in all treated groups and higher in BMSCs than HUCSCs group	[101]
Rat	Sciatic nerve	Nerve growth factor "NGF"	Removal of 15 mm of nerve	0.05 mg/mL of NGF	PLGA conduit	Nerve Histomorphometry, Motor Endplate Evaluation and Gastrocnemius Muscle evaluation	1-On 21st day, there were no changes in gastrocnemius relative muscle mass ratio differences in both groups. 2-At 180 days the NGF conduit group had significantly high muscle mass than the non-drug group.	[102]
Rat	Sciatic nerve	Rat dermal fibroblasts (RDFs) + human umbilical vein endothelial cells (HUVECs)	Removal of 8 mm nerve with 10 mm gap	5 x10^5^ of HUVECs and 5 x10^4^ of RDFs	Silk fibroin poly l-lactide-co-ε-caprolactone “SF/P(LLA-CL)” and poly l-lactide-co-ε-caprolactone alone “P(LLA-CL)”	*In vivo* evaluation of vascularization and nerve regeneration by immunohistochemistry	1-At 3 weeks after implantation, the blood vessel area in the SF/P (LLA-CL) group (5.23±0.87%) was higher than that in the P(LLA-CL) group 3.61±0.97% 2-Schwann cells and axons, S-100, and NF-200-positive areas in SF/P (LLA-CL) group was more than P(LLA-CL) group.	[103]
Rats (adult female Sprague Dawley)	Sciatic nerve	Neural crest cells differentiated from human embryonic stem cells (hESCs)	Removed 5 mm of nerve with a 10 mm gap	One million cells	Tubular conduit made from Trimethylene carbonate ε‐caprolactone block‐copolymer	Histology and immunohistochemistry and Image analyses	1-The transplanted NCCs were closely linked to the developing axons, and certain subsets further differentiated into glial-like cells in the conduit environment, demonstrating a strong regeneration throughout the conduit lumen.	[40]
Rat	Sciatic nerve	Gingival derived mesenchymal stem cells (GMSCs)	The nerve crushed for 30	2 × 10^6^/mL GMSCs	3D-collagen hydrogel	Electro-physiological analysis, sciatic functional index (SFI,) Immunohistochemical studies & Morphological evaluation	1-At 4 weeks post-injury, NP/GiSC had significantly better effects than NP alone on the percentage of conduction velocity or recovery of motor nerve conduction velocity 2-The myelin sheaths of the NP/GiSC group were even thicker than those of the empty NP group	[42]
Mice (Male Sprague Dawley)	Sciatic nerve	rodent and human skin-derived precursors (SKPs) + SKP-derived Schwann cells	Crushing of nerve for 1 min	1–4 x10^5^ cells injected into the distal nerve	Without conduit	electron microscopy (EM) and immunocytochemistry	1-2 weeks after transplant, many YFP-labelled cells associated with neurofilament-positive axons were observed “in case of murine SKPs”. 2-At 6 weeks after transplantation of SKP-derived Schwann cells, about 26,000 YFP-positive cells had integrated into this 1.5 cm segment of the nerve.	[104]
Mice (SCID)	Sciatic nerve	Muscle-derived stem/progenitor cells (MDSPCs) isolated from adult human skeletal muscle (hMDSPCs)	Removal of 4 to 5 mm of the nerve	4 × 10^5^ of (hMDSPCs) was injected at the proximal and distal nerve stumps	Without conduit	Immunohistochemistry, Morphometric analysis of the regenerated nerve, Muscle reinnervation, and Functional assessment	1-12 weeks after injury, the group with hMDSPCs showed remarkable improvement in their capacity to sustain the wounded leg at the same level as the uninjured leg. 2-72 weeks after injury, the weight of gastrocnemius muscles of the hMDSPCs regain normal weight.	[61]
Mice	Tibial nerve	Embryonic stem cell-derived motoneurons “ESCMNs”	Transaction of nerve followed by ligation inside medial gastrocnemius “Proximal stump”	Ten thousand cells in 0.1 μL were transplanted in the distal nerve stump + 10 μm serotonin hydrochloride (5-HT)	Without conduit	*In vitro* electrophysiological examination of medial gastrocnemius muscle	1-3 months after transplantation Some rats exhibit contraction of the muscle upon electrical stimulation of the transplant site. 2-Other rats were spontaneously rhythmic in the absence of electrical stimulation 3- addition of NMDA, and 5-HT “neurochemicals” cause enhancement of rhythmic motor output of the muscle	[105]
mice (FVB)	Sciatic nerve	Neural stem cells (NSCs) + IL12p80	Removal of 3 mm nerve segment	1 × 10^6^ NSCs and/or 100 ng mouse IL12p80	micropatterned poly (L-lactic acid) (PLA) conduit	Walking track analysis and Rotarod test, Immunofluorescence staining, Hematoxylin and Eosin (H&E) staining and immunohistochemistry staining, and CMAP analysis	1-On the eighth week, Mice of Conduit+mIL12, Conduit+NSC, and Conduit+NSC+mIL12 groups showed higher SFI score than the Conduit only. 2-The CMAP in The Conduit+NSC+mIL12 group showed better recovery status than other groups	[106]
Mice	Sciatic nerve	Human embryonic stem cells (HESC)	5 mm long segment removed,	3 × 10^5^ HESCs were injected at the site of injury	Heterologous fibrin sealant (F)	Immunohistochemistry, Catwalk test & von-Frey test	60 days post-surgery, 1-The highest integrated density of anti-VGLUT1 antibody "marker of sensory neuron" was observed in N + F + D + T group. 2-The best scores of SFI belonged to the groups that incorporated HESCs	[107]
Rabbit	Peroneal nerve	Autologous Schwann cells	A 60 mm segment of the nerve was transected	Schwann cells resuspended in Matrigel at 10^6^/mL concentration	Autologous vein nerve conduit (6 cm of the gluteal vein) (AVNC)	Light and electron microscopic measurements	1-Four months postoperatively, axonal and myelin components were more detectable in the Schwann group than control. 2-There were myelinated fibers in distal-most sites in the Schwann group but small myelinated fibers and no axons in the same site for the control group.	[108]
Rabbit	Tibial nerve	PRP + Nerve micro-tissue (Micro-T) + and previously prepared Schwann cells (SCs)	Removal of 12 mm from nerve	12 mm long tibial nerve divided into three equal parts (4-mm/part) to make (Micro-T) + 300μl PRP + 1.5× 10^4^ SCs	Autologous vein nerve conduit (18-mm-long subcutaneous vein)	Nerve function evaluation, ultrasonography, Quantitative real-time RT-PCR, Electrophysiological recovery evaluation & Morphological evaluation	1-Functional scores (FSs) between the Micro-T+PRP and Autograft groups did not significantly differ at 12 weeks following surgery. 2-The ratio of CMAP amplitude and the ratio of CMAP latency were significantly improved in the Micro-T+PRP group.	[109]
Beagle dogs	Common peroneal nerve	basic fibroblast growth factor (bFGF)	Removal of 3 mm nerve segment	500 μL of bFGF in 0.5-mL gelatin hydrogels	5-cm-long A cellular allogenic nerve graft “Sciatic nerve graft”	Immunohistochemistry, Histology and Electron Microscopy and Electromyography	1-One Month after Grafting, an abundance of regenerating axons was found in auto- and allografts with bFGF treatment 2-3 months after Grafting, the no. of regenerating axons was higher in the autografts “22.6/104 μm2 “than the other bFGF-treated allografts	[38]
Dog	Ulnar nerve	Autologous dermal fibroblasts	Removal of 5 mm segment of nerve	3 × 10^5^ cells/mL	An 8-mm Bio 3D conduit and silicone tube with 8 mm length and 2 mm internal diameter	Pinprick Test, Histological and Morphometric Studies, Electro-physiological Studies, Immuno-histochemistry, and Wet Muscle Weight	Ten weeks after surgery, 1-The mean wet weight of the hypothenar muscle was 0.95+0.14 2-The mean MNCV was 34.6+7.0 m/s in the Bio 3D group 4- PPT scored as grade 3 for 4 affected forearms.	[41]
Pig	Ulnar nerve	Autologous Schwann cells	Transaction of about 50mm long segment	10 × 10^6^ Schwann cells were injected evenly along the nerve graft	Ulnar nerve allograft	Histological Evaluation and Quantitative Histomorphometry evaluation	1-20 weeks after transplantation, MHC-matched Schwann cells allografts showed mean fiber counts of 9,195 ± 4,061 compared to allografts with saline 3,924 ± 932 (*P* < 0.05). 2-Pretreatment with UV-B-irradiated donor alloantigen did not show a benefit for nerve regeneration	[110]
Mini pig	Sciatic nerve	Human amniotic fluid stem cells (HAFSCS)	Transection of nerve leaving 15 mm gap	6 x10^6^ AFSCs	2.5 cm PLA	Measurement of compound muscle action potential (CMAP) of tibialis anterior (TA) muscle and extensor digitorum brevis (EDB), Conventional MRI and diffusion tensor MRI and RT-PCR	1-At the 16th month after surgery, the conduit + HAFSCs group's CMAP of TA performed better than the conduit-only group and also had a higher ratio of regenerated sciatic nerve fibers 2- RT-PCR studies suggested that AFSCs may Possess characteristics resembling those of neural stem cells.	[111]
rhesus monkey	Ulnar nerve	Autologous non-hematopoietic mesenchymal stem cells (MSCs)	40 mm Removed from the nerve	2×10^7^ cells of MSCs & 2×10^7^ cells of SCs	A cellular nerve allograft	Electrophysiological assessment of reinnervation, Neurofilament immunohistochemistry, and axon quantification	1-6 months following grafting, The mean peak CMAP and the mean NCV of the non-implanted group was lower than allografted groups (*P*<0.05) 2-The mean numbers of the NF-positive fibers were highest in both cellular allografted groups but lower in the non-implanted group	[112]

SC group “Schwann cell group”, pfu “plaque forming unit”, NCV “nerve conduction velocity”, NP/GiSC “nerve protector/gingival Schwann cell”, YFP-labelled “yellow fluorescent protein-labeled”, NMDA, 5-HT “N-methyl-D-aspartate receptor, 5-hydroxytryptamine receptors”, mIL12 “mice interleukin 12”, anti-VGLUT1 “anti-Vesicular glutamate transporter 1antibody”, N + F + D + T group “Neurorrhaphy + heterologous fibrin sealant + hESCs on (doxycycline)”, MNCV “motor nerve conduction velocity”, PPT “Pinprick Test”, MHC-matched “major histocompatibility -matched”, UV-B “ultraviolet B”, FK506 “

**Table 7:** Illustrate miscellaneous types of nerve grafting therapy research

Animal	Nerve involved	Type of nerve graft	Type of injury	Fabrication method	Length of conduit	Clinical evaluation	Clinical results	References
Sprague–Dawley transgenic rats	Sciatic nerve	Acellular nerve allografts (ANAs)	Transaction of the nerve 20, 40, and 60 mm	Autografting followed by decellularization	20, 40 and 60 mm long	Histomorphometry, Functional assessment, Histological analysis, Quantitative reverse transcriptase PCR (qRT-PCR), and Electron microscopy	1-At 10 weeks, it was shown that in both graft groups, longer grafts had lower rates of axonal regrowth. 2-At 20 weeks, there were significantly fewer nerve fibers in the 40 mm ANA compared to the isograft, and no fibers were detected in 60 mm ANAs grafts	[113]
Rat	Median nerve	Bi-layer chitosan Membranes " Flexible nerve guidance channels (NGCs)"	Removal of about 10 mm	Solvent casting	12 mm length with a 1.1 diameter	A combination of Immuno-histochemical " confocal laser microscopy" and histological investigations " Resin embedding and transmission electron microscopy	1-After 12 weeks following surgery, bi-layer membranes showed a high population of NF axon alignment. 2-Typical regenerated nerve fibers were found in both experimental groups.	[114]
Rat	Median nerve	Chitosan cross-linked with DSP (CS/DSP) alone or in association with GPTMS (CS/GPTMS)	5 mm of the nerve was removed	Solvent casting Technique	10 mm long	Immunohistochemistry, confocal laser Microscopy, Resin embedding and electron microscopy & Design-based quantitative morphology of nerve fiber regeneration	12-week post-injury: 1-Total number of myelinated fibers was (autograft = 6916 ± 1633; CS/DSP = 7249 ± 113). 2- Axon and fibers diameters and *G*-ratio were significantly lower (*p < 0.05) in CS/DSP group than the autograft group 3-no more significant differences were detectable between autograft and CS/DSP group in functional recovery of the median nerve	[115]
Rat	Sciatic nerve	Poly carpo-lactone (PCL) and silicone tube	Removal of 16mm and 10mm of nerve	Dip‐coating, salt leaching Technique	1.8 cm long PCL and 1.2 cm long silicone tube	Micro-CT imaging of PCL conduit and silicone tube at 14 weeks and 6 weeks survival	1-Micro-CT imaging of the PCL conduit showed it surround by lighter inflammatory cells and new blood vessels. 2-Axons were detectable inside and outside the conduit. 3-Micro-CT imaging of the silicone tube confirmed the lack of intact tissue between the stumps.	[116]
Rat	Sciatic nerve	Polycaprolactone (PCL) conduit + Nap-FFGRGD	A segment of the nerve was removed, leaving a 15 mm gap	Electrospinning	17 mm long	Immunofluorescent Staining Assay, Morphometric Analysis, FG Retrograde Tracing, Electrophysiological Assessment, and histological studies	1-At 12 weeks post-surgery, the SFI in the RGD-PCL and YIGSR-PCL groups was significantly higher than those in other groups indicating that RGD and YIGSR can perform better motor functional recovery	[117]
Rat	Sciatic nerve	3D-engineered porous conduit of gelatin cryogel	Transaction of 5 mm	Indirect 3D printing	A length of 1cm, an inner diameter of 1.5 mm, and an external diameter of 4 mm	Walking track analysis, Electrophysiology, Histological evaluation, and Immunohistochemical evaluation**.**	1-2 months after surgery, there was an intense and abundant positive reaction in the conduit group (15.44%) which was larger than in the end-to-end group (8.61%). 2-Animals in the conduit group attained a mean SSI value of “− 47.26 ± 0.36” which was “− 62.97 ± 1.1” in the end-to-end group	[118]
Rat	Sciatic nerve	*in vivo* created vascularized neurotube “Silicone rod”	Removal of 10 mm of nerve	Commercial	1.5 cm silicone rods with 1 mm in diameter	Electrophysiological evaluation, Histomorphometric evaluation, and Gross examination	1-The vascularized conduit group had significantly improved the mean peak amplitudes “15,24 _ 2,76 mV”. 2-The myelinated axonal counts of the vascularized conduit group were higher than other groups “65,93 _ 11,41”. 3- No adhesion and scar formation were observed in groups of vascularized conduits	[119]
Rat	Sciatic nerve	Oxidized polyvinyl alcohol (OxPVA), neat polyvinyl alcohol (PVA) and silk-fibroin (SF)	Removal of 5 mm of nerve	Mandrel‐coating Technique	10 mm in length	Histological and immunohistochemical analysis, Morphological and morphometric assessment of nerve regeneration, and Gait analysis	1-The animals recovered their gait after 12 weeks, however, the PVA group's paws performed worse than the others 2-In comparison to PVA and SF, the reversed-autograft ensured higher results in terms of total axons/nerve in the central part.	[120]
Rat	Sciatic nerve	Freeze-cast porous chitosan conduit	Removal of 10 mm nerve	Electrospinning	12 mm long	Histomorphometry analysis through H&E and IHC staining of longitudinal and transverse sections.	1-NF-staining show that the proximal, middle, and distal portions of the conduit all had longitudinally directed regenerated nerve fibers. 2-In the conduit lumen well-vascularized cellular connective tissue surrounding the neuron and the nerve itself, all are observed.	[121]
Sprague Dawley male mice	Sciatic nerve	A poly-lactic-co-glycolic acid (PLGA) nerve conduit	Removal of 5 mm of nerve	Molding	5 mm in length	Measurement of biostability of the device to support axonal growth through 3 weeks old period	1-After three weeks, the inflammatory response was seen to be equivalent to areas without any conduit 2-The device can sustain functional stability for three weeks and would not result in any substantial inflammatory reaction	[122]
Zealand white rabbits	Peroneal nerve	Autogenous vein nerve conduit (AVNCs)	Removal of 1,3 and 6 cm long segments of nerve	Autografting	A length of up to 6 cm of AVNCs of the left gluteal vein	Electromyography (EMG), motor nerve conduction studies and neurophysiologic examination	1-At the 6-month, all animals in AVNC Groups 1 and 2 showed signs of moderate to abundant nerve development into the distal nerve segment through the nerve conduit. 2-axonal regeneration, with excellent myelinization and organization of the distal nerve	[123]
Female New Zealand white rabbits	Motor branches of vastus medialis & rectus femoris muscles	Saphenous nerve graft	Cutting of motor nerve	Autografting	3 cm, 5 cm and 7 cm long	Gross findings, Measurements of muscle force, and Morphometric analysis	1-Rectus femoris muscle showed an average maximum tetanic force of 27.2 N. 2-Average number of myelinated fibers in the distal end of the graft was 2283.	[124]
New Zealand white rabbits	Sciatic nerve	Multi-channeled scaffold characterized by aligned electrospun nanofibers and neurotrophic gradient “MC/AN/NG”	Removal of 15 mm of nerve	Electrospinning	The 15 mm-long scaffolds were fitted with the proximal and distal nerve ends	Electrophysiological tests, relative gastrocnemius muscle weight (RGMW), and histological analysis	1-The amplitude of CMAP and the NCV values were detected after 8, 16, and 24 weeks in all groups 2-At 16 and 24 weeks, the amplitude of CMAP and the NCV values of the MC/AN/NG group were higher than non-grafted group. 3-Axon diameter, the thickness of myelin sheath, and the diameter of myelinated fibers were better in the MC group than SC group	[125]
Cat	Sural nerve	Autologous nerve graft (The removed nerve segment)	Removal of 20 mm and 30 mm nerve segment	Autografting	20 mm and 30 mm long	Electrophysiological assessment & histological measurements	1-one year after surgery, The % of unidentified afferent fibers was higher than the control group. 2-The NCV distal/proximal stumps ratio was as follows: 20 mm autograft….42% 30 mm autograft ….51%	[126]
CAT	Sciatic nerve	Alginate sponge with or without tubulation	Removal of 50 mm of nerve	Freeze drying	Alginate sponge coated with polyglycolic acid mesh” was sutured to the two nerve stumps	Electrophysiological Evaluation, Histological Analysis, and Morphometric Analysis	1-At 3 months after surgery, CMAP and SEP showed remarkable restoration in both the tubulation and non-tubulation groups. 2-The axonal density of myelinated fibers was the same in the two groups, but regenerated axons showed less diameter and higher density than normal	[127]
Beagle dogs	Common peroneal nerve	Polyglycolic acid (PGA)–collagen tube	Removal of 80 mm nerve segment	Enzymatic digestion of pig skin to obtain collagen	90 mm in length.	Histological evaluation, Immunohistochemistry, Morphometrical analysis, and Electrophysiological recording	1-Locomotor function appeared to be nearly normal in the majority of dogs after 10–12 months. 2-S100- protein immunoreactivity was seen at the regenerated segment at 12 months “representing Schwann cells ensheathing and encapsulating in the regenerated axons.	[128]
Dog	Sciatic nerve	chitosan/PGA graft	Resecting a 26 mm segment of nerve, leaving a 30 mm gam	Freeze drying	30 mm long	Electrophysiological assessment, Histological assessment, Fluorogold retrograde tracing, Immuno-histochemistry, EM &Morpho-metric analysis	1-The chitosan/PGA graft dog's typical conduction velocity was 33.5% of that of the control. 2-Masson trichrome staining of gastrocnemius muscle shows clear cross-striations on longitudinal sections. 3-% of NF immune-Positive areas and myelinated axon density of the chitosan/PGA graft dog were higher than those for either the autograft dog or the normal dog.	[129]
Adult Beagle dogs	Sciatic nerve	Longitudinally oriented collagen conduit (LOCC) combined with nerve growth factor (NGF)	Removal of 35 mm of nerve	Freeze drying	40 mm long	Immunohistochemistry, Luxol fast blue staining analysis, Electron microscopy analysis, and Evaluation of gastrocnemius muscle function	1-The CMAP ratios and the muscle weights in the proximal and distal parts of the injured sciatic nerve were in the following order “9 months after grafting “Autograft group> LOCC/NGF group> LOCC group>vehicle group.	[130]
Rhesus Monkey	Median & Ulnar nerves	Collagen nerve guide and polylactate nerve guide + Autografting	5 mm long removed	Solvent casting	5 mm long	Electrophysiological measurements & Morphometric analyses.	1-At 56 d post-surgery, there were no significant differences in the average time of CMAP & CSAP between nerve guide & graft groups. 2-By 800 days the recovery of motor and sensory amplitudes reached to be stable then increased to be similar to normal	[131]
Outbred ewes	Median nerve	Autografts (median) and allografts from the radial nerve with a cyclosporin immune suppression	Removal of about 50 mm of nerve	Autografting and allografting	8 cm of radial nerve allograft and 5 cm of median autograft	Hematoxylin and eosin (H&E) and Luxol fast blue (LFB) and immunostaining sections were taken for evaluation	1-From 35 to 47 days after surgery, Nerve regeneration was better in the proximal and distal segment in both allograft and autograft with immunosuppression 2-No regenerated axons were seen in the distal segment in the allograft without immunosuppression	[132]
Sheep	Tibial nerve	Decellularized vein grafts filled with spider silk fibers	Transaction of 60 mm segment of nerve	Autografting	A length of 6 cm of Venules taken from veins of the lower extremities	Electrophysiological recordings, histology, and morphometric analysis for axon counting	1-10 months after surgery, Transplantation of autologous nerve resulted in axonal regeneration followed by myelination. 2-No significant difference in the CMAP and MNCV between autografted and construct groups at 10 months	[133]
Merino breed sheep	Common peroneal nerve	Nerve guidance conduit (NGC) (Reaxon®)	Neurotmesis and Axonotmesis	Commercial	3 cm long and 3.0 mm in diameter	Neurological Evaluation, Nerve Morphological, and Stereological Analysis	1-No clinical improvements were observed until week 4 for all the therapeutic groups 2-The axonotmesis group completely recovered their postural balance after 12 weeks. 3-At 24 weeks, the NGC group performance exceeded the animals with End-to-end sutures	[134]

DSP “dibasic sodium phosphate”, GPTMS “γ-glycidoxypropyltrimethoxysilane”, Nap-FFGRGD “Naphthalenephenylalanine-phenylalanine-glycine-arginine-glycine-aspartic”, RGD-PCL “arginine-glycine-aspartic- polycaprolactone”, YIGSR-PCL “tyrosine-isoleucine-glycine-serine-arginine- polycaprolactone”, SSI value “static sciatic index”, MC group “multi-channeled scaffold group”, SC group “single-channeled scaffold group”, CSAP “compound sensory action potential”.

**Table 8:** Illustrate miscellaneous types of therapeutic medicine approach research

Animal	Nerve involved	Type of medicine	Type of injury	Dosage	Route of application	Clinical evaluation	Clinical results	References
Rat	Sciatic nerve	Cytokine lymphotoxin (LT)	Crushing of 5 mm of nerve	20 microgram /kg every 24 hr. and 1 hr. before the surgery	Intraperitoneal injection	Walking track test, Histological examination, & Motor Functional Assessment	1-In the LT pretreated group, motor functional recovery started on day 7. 2-During the 2nd week following the crush, the SFI in the LT group improved more quickly than in the controls, and on day 18, the difference was significant (*P* <0.05): − 30.9 + 7.2	[135]
Rat	Sciatic nerve	methanolic extract of Ocimum sanctum As nerve tonic”	Transaction of 5 mm	Different doses (50, 100, and 200 mg/kg )for 10 consecutive days	Orally	Behavioral studies and pharmacological studies, Biochemical estimation of markers of oxidative stress, and Histopathological analysis	1-Following nerve transection, a high dose of Ocimum sanctum causes attenuation in decreased motor performance. 2-At 14th post administration, Ocimum sanctum led to attenuation of axotomy-induced rise in TBARS, total calcium, and decrease in GSH levels in a dose-dependent sequence.	[136]
Rat	Sciatic nerve	Zofenopril "Antioxidant agent"	Crushing of nerve for 30 seconds	15 mg/kg/day for 7 days	Zofenopril was administered orally	Walking track analysis, Motor nerve conduction velocity (MNCV) & Morphological analysis	1-On 14th day post-surgery, SFI and (EMG) studies significantly differ from one group to another 2-On 42nd day post-surgery, no significant difference between groups.	[137]
Rat	Sciatic nerve	Alfa Lipoic acid (a-LA) " Neuro-protective metabolic antioxidant"	Crushing of nerve for 60 seconds	100 mg/kg of a-LA at 24 hr. and one hr. before crush injury.	Intra-peritoneal Injection	Evaluation of biochemical parameters " CAT, SOD& MDA activities "	1-At the first hour (Group III, *p* 0.05) and third day (Group IV, *p* 0.05) following a-LA therapy 2-Tissue SOD and CAT activities improved, and the MDA level reduced significantly	[138]
Rat	Sciatic nerve	Propolis and curcumin “antioxidant” + Methyl- Prednisolone “steroid “	Crushing of nerve for 30 seconds	100 mg/kg curcumin, and 200 mg/kg propolis Starting from the day of surgery for 28 days later	Orally through nasogastric intubation	Walking track analysis and electrophysiological measurements, histomorphometric, electron microscopic, and muscle weight measurement	1-At 4 weeks for SFI, Cu Group, and Pr Group had statistically better values than the CM group. 2-Amplitude values of S, CM, Cu, and Pr groups were better than the control group. 3-no significant difference between groups in gastrocnemius muscle weight	[139]
Rat	Sciatic nerve	Dexamethasone Drug “DM”	Clamping of the left sciatic nerve for 60 s using pincers with a 2 mm width	0.5 mg/kg in group(c), 1 mg/kg in group(d), 2 mg/kg in group(e)	Local intramuscular injection for 10 days	Sciatic functional index, gastrocnemius muscle mass ratio, immunohistochemistry & histological evaluation.	1-SFI values rebounded to −58.15 ± 3.1, −56.56 ± 1.7, and −56.07 ± 3.5, respectively, in groups (c), (d), and (e), and were significantly higher than group b “control” 2-Histological sections show that total myelinated axon no. decreased due to crushing that was attenuated by I/m injection of DM.	[140]
Rat	Sciatic nerve	Sildenafil Citrate	Crushing of nerve for one minute.	“Group 1" received 20 mg/kg daily “Group 2” received every-other-day 10 mg/kg	Orally via a nasogastric tube	Rotarod and the accelerod tests, Static sciatic index, Hot plate test, Measurement of Bone Mineral Density (BMD) and Histopathological evaluation	1-When comparing the treated group to the control one in the 4-min test, the control group took less time to stay on the rod for the duration of the accelerod test. 2-6th weeks post-surgery, there were no significant differences between the groups in SFI.	[141]
Rat	Sciatic nerve	17-beta-estradiol "Estrogen hormone "	Transaction of about 3 mm	10 μL a of 17- beta-estradiol (0.1 mg/mL)	Silicon conduit filled with estradiol was inserted at the lesion site by epineural suture	Static Sciatic Index (SSI), Behavioral Testing, Electrophysiological Assessment, Biomechanical Testing, Muscle Mass and Histological Preparation and Morphometric Studies	1-At 12 weeks, the SFI estradiol group was higher than in the silicone group. 2-The muscle weight ratio in the Estradiol group was higher than in the silicon group. 3-The Estradiol group displayed larger nerve fiber, axon diameter, and myelin sheath thickness than the silicone group.	[142]
Rat	Sciatic nerve	Laser therapy and swimming exercise protocols	Crushing of 5 mm s of nerve for 60 seconds	The laser was applied at 1st, 2nd, 3rd &4th week & swimming exercises were performed five times a week	Irradiation was performed on the affected limb for 8s 16s, 24s.	Functional (FCI) evaluation and the nerve morphometry (areas, diameters, and thicknesses of the fibers, axons, and myelin sheath)	1-Compared to the SCG and ELSG groups, the area and diameter of the axon from the ELG and ESG groups had the best results. 2-The functional analysis results from the ELSG group were better than the other groups	[143]
Rat	Sciatic nerve	Cerebrospinal fluid (CSF) in a collagen guide channel	Removal of about 10 mm of nerve	Not recorded	CSF was injected within the collagen conduit	Sciatic functional index (SFI) and electrophysiology, histology, and immunohistochemistry testing.	1-At 49th & 60th day after surgery, SFI of the collagen +CSF group was significantly higher than the autograft group (*P*< 0.05) 2-At 90 days after surgery, nerve conduction velocity (NCV) of the collagen +CSF group was greater than autograft group (*P*< 0.05)	[144]
Rat	Sciatic Nerve	Methyl cobalamin (MeCbl)	Crushing of nerve for 10 second	10 μL 3% MeCbl	MeCbl was injected in a nanofiber sheet (10 × 10 mm)	Plasma concentration of MeCbl, Sciatic Function Index & von Frey filament test, Electrophysiology, and Histology	1-6 weeks after the operation, The SFI value in the MeCbl group was significantly higher than that in the untreated group. 2-Values of NCV were faster in the MeCbl local (44.4 ± 2.8 m/s) and MeCbl systemic (43.2 ± 2.5 m/s) groups compared to untreated (28.2 ± 2.5 m/s)	[145]
Rat	Sciatic nerve	Low-intensity ultrasound (LIU)	3 mm of the nerve was crushed for 30 sec.	1 MHz frequency, 0.2 W/cm^2^ intensity of (LIU) Starting 72 h after surgery, then every other day " 1 min/day	(LIU) was directed to the crushed site of the nerve	Catwalk gait analysis, Electrophysiological test, Wet weight ratio of the target muscle, Electron microscopy, and Quantitative real-time PCR	1-At 2,3 & 4 wk. after surgery, SFI values and CMAP in the treatment group were higher than in the control group. 2-BDNF mRNA expression increased over time, from 1,2,3, and 4 weeks in the treatment group. 3-The treatment group had higher wet-weight ratios of the gastrocnemius muscle.	[146]
Rat	Sciatic nerve	Genistein and Gabapentin	Crushing of nerve for 60 s. + Transaction of nerve	Genistein "20 mg/kg" and Gabapentin" 90 mg/kg" for 30 days.	Intraperitoneal injection	Immunohistochemical analysis, Measurement of pro-inflammatory cytokine level in sciatic nerve, Walking Track analysis, sciatic function index (SFI) and Paw mechanical withdrawal threshold measurement	1-GAP-43 and MBP immunoreactivity in the Genistein groups were higher than in other groups. 2-The levels of IL-1 and TNF- were not significantly different between the Genistein and gabapentin groups 3-A statistically significant change in SFI was seen at 4th between the control and all treatment groups	[147]
Rat	Sciatic nerve	Simvastatin in Pluronic F-127 hydrogel	Removal of 10 mm of nerve	20 μL of Pluronic F-127 hydrogel containing different doses of simvastatin (0, 0.5, or 1.0 mg)	Simvastatin was injected into the hollow chitosan conduits which	Walking track analysis, Electrophysiology, Retrograde tracing with Fluro-Gold, Histological and immunohistochemical evaluation, Transmission electron microscopy, and Gastrocnemius wet weight	1-Beginning at 4 weeks, the SFI values in treated groups were higher than the control group “*p*<0.05” 2-The numbers of NF200-positive cells and S100-positive cells were significantly larger than in the conduit group. 3-The CMAPs and MNCV values were higher in the treated groups than control (*p*<0.05)	[148]
Mice (adult male ICR)	Sciatic nerve	Mecobalamin “a form of vitamin B12”	Crushing of 2 mm of nerve for 30	(A low dose “65 **μ**g/kg” and a high dose “130 **μ**g/kg”)	Intraperitoneal injection of	Walking track analysis, Micromorphological examination, and Real-time PCR	1-At 15 and 20 days, the sciatic functional index was higher in both mecobalamin groups than in the saline group (*P* < 0.01). 2-thickness of myelin sheath formed in regenerated axons was higher in the high-dose mecobalamin group than in the saline group (*P* < 0.05).	[149]
male inbred miniature swine	Ulnar nerve	Injection of FK506 Immune suppressant molecule tacrolimus	Removal of 80 mm segment of nerve	0.1 to 0.4 mg/kg of FK506	I/V injection and local injection at the site of injury	Histomorphometric Analysis	1-At 24 weeks post-transplant, Strong nerve regeneration occurred distal to autografts in treated animals with FK506. 2-No evidence of nerve regeneration was present in mid-graft sections from allografts and untreated animals and low no. in distal nerves than in autograft groups	[150]

TBARS “Thiobarbituric acid reactive substance”, GSH “Glutathione”, EMG “electromyograph”, SOD “superoxide dismutase”, CAT “catalase”, MDA “Malondialdehyde”, Cu group “Curcumin Group”, Pr group “Propolis Group”, CM group “Control-Methylprednisolone Group”, S group “Sham Group”, TNF “tumor necrosis factor”, FK506 “macrolide immunosuppressive drug”

## Data Availability

All data collected or analyzed during this study are included in this published review.

## Abbreviations

CNS: Central nervous system
NCS: Neural stem cells
PNI: Peripheral nerve injuries
GMSCs: Gingival-derived mesenchymal stem cells
MSCS: Mesenchymal stem cells
bFGF: Basic fibroblast growth factor
BMSCS: Bone marrow mesenchymal stem cells
GDNF: Glial cell line-derived neurotrophic factor
ADSCs: Adipose-Derived Stem Cells
NGF: Nerve growth factor
OECs: Olfactory ensheathing cells
NTF3: Neurotrophin 3
BDNF: Brain-derived neurotrophic factor
PCL: Polycaprolactone nerve conduit
GDNF: Glial cell-derived neurotrophic factor
AdBMP7: Gene transfer of adenoviral bone morphogenetic proteins
PGA: Poly glycolic acid
